# An Unrealistic Drift in Assay on Anhydrous Basis towards Content Limit

**DOI:** 10.4103/0250-474X.59553

**Published:** 2009

**Authors:** K. Shivram, D. M. Patil, Nitu D. Shah, Riddhi U. Thite, A. S. Joshi, N. P. Motka

**Affiliations:** Analytical Development Division, Sun Pharma Advanced Research Company Limited, Tandalja, Vadodara-390 020, India

**Keywords:** Assay on as-is basis, assay on anhydrous basis, propagation of errors, standard deviation

## Abstract

The assay on anhydrous basis is a mathematically derived value from an experimental results of assay and water content tests. The results of assay and water content tests are determined, separately, on as-is basis. The industry-accepted formula for assay on anhydrous basis = (assay on as-is basis×100)/(100-%water). Statistically, the two variables involved in accepted formula are assay on as-is basis and water to obtain assay on anhydrous basis. The experimental errors associated with these two variables propagate in assay on anhydrous basis. The error propagates either in constructive or destructive mode. The constructive mode of error propagation is combination of positive error of assay on as-is basis and positive error of water or negative error of assay on as-is basis and negative error of water. The constructive mode of error propagation has more impact on assay on anhydrous basis values and its confidence interval. The destructive mode of error propagation is combination of a positive error of assay on as-is basis and a negative error of water or vice versa. The destructive mode of error propagation has lesser impact on assay on anhydrous basis values and its confidence interval in comparison to the constructive mode of error propagation. In accepted formula said above, the constructive or destructive error propagation causes unrealistic drift of assay on anhydrous basis towards either lower or higher side of content limit of substance. The risk of rejection of pharmaceutical use substance is higher based on assay test results that results are calculated from industry-accepted formula. The purpose of the study is to propose an alternative formula to overcome limitations of accepted formula and justify the propagation of errors in realistic way. We have given three examples of pharmaceutical use substances to emphasise the above proposition. The proposed formula for assay on anhydrous basis= (assay on as-is basis×Φ)/(Φ-%water) in which Φ is sum of experimental results of assay and water content tests experimentally determined, separately, on as-is basis.

The content limit for assay test in almost all monograms of pharmaceutical use substance in several pharmacopeias is defined on anhydrous basis. In routine analytical practices, the assay test of pharmaceutical use substance is being performed without rendering to anhydrous state. The result of assay test is termed as assay on as-is basis. The water present in a pharmaceutical use substance is not considered as an impurity and hence the result of water content test is accounted in the result of assay on as-is basis. The water is accounted in assay on as-is basis mathematically by using industry-accepted formula for assay on anhydrous basis. The industry-accepted formula is written as (assay on as-is basis×100)/(100-%water) and out come of formula is termed as assay on anhydrous basis[1].

The basis for industry-accepted formula is a chemical mass balance method. According to chemical mass balance method, % total theoretical mass of chemical substances present in a mixture is 100. For example, the theoretical weight percentage of sodium citrate dihydrate is sum of weight percentage of sodium citrate (87.8%) and weight percentage of water content (12.2%). In industry-accepted formula, it is assumed that sum of content of % sodium citrate (AAI) and % water content (W) is equal to 100[2]. The theoretical mass balance equation is written as AAI+W=100 (Eqn.1), where AAI is assay on as-is basis and W is water. Usually, the experimental values of AAI and W are deviated to either positive or negative side from theoretical values. The deviation of AAI and W from theoretical value is considered as error (E). The experimental value of AAI and W is denoted as AAI±E_AAI_ and W±E_W_. The Eqn.1 is modified for experimental value of AAI and W as (AAI±E_AAI_)+(W± E_W_)= 100 (Eqn. 2). Mathematically the path followed for propagation of error in industry-accepted formula for assay on anhydrous basis is given as (AAI×100)/(100-W)= 100±[(E_AAI_±E_W_)×100/(100-W)](Eqn.3). The value AoA_a_ cannot be 100% in Eqn. 3 because the term (E_AAI_+E_W_) is never zero. In alternate formula, assay on anhydrous is calculated by substituting 100 by ‘Φ’ in industry-accepted formula and ‘Φ’ is sum of experimental results of assay and water content tests determined. Mathematically the path followed for propagation of error to assay on anhydrous basis in alternate formula is [AAI×Φ]÷[Φ-W]=100±[E_AAI_+E_W_] (Eqn.4). The value AoA_p_, in Equ.4, is function of sum of errors associated with assay and water content only.

The assay on anhydrous basis calculated using industry-accepted and alternate formula is denoted as AoA_a_ and AoA_p_, respectively. The drift (ΔAoA) is a deviation of AoA value from 100 i.e. ΔAoA= |100-AoA|. The ΔAoA for industry accepted and alternate formula are denoted as ΔAoA_a_ and ΔAoA_p_, respectively. The relation between ΔAoA_p_, ΔAoA_a_ and water is ΔAoA_a_=ΔAoA_p_× [100/(100-W)] (Eqn.5). It is clear from Eqn.3 that the unrealistic propagation of errors in AoA_a_ calculation is not being considered in setting assay limit[3].

Almost all substances of pharmaceutical use described in pharmacopeias have water content below 30% w/w. The substances containing water from 5% to 30% is grouped in level six for simulated model-1 preparation and difference in water between two successive levels is maintained to 5%. The values of AAI and water are termed as ideal values. The simulated model-1 is designed to understand the propagation of inaccuracy error associated with AAI and W to AoA. The ideal values of AAI and W is deviated by ±1%. The constructive mode of error propagation is set by deviating (−1%) and (+1%) the ideal value of AAI and W for first and second group, respectively. The destructive mode of propagation is set by deviating (+1%) of ideal value of AAI and (−1%) of ideal value W for third group or vice versa for fourth group. The values AoA_p_ and AoA_a_ are calculated from deviated data of AAI and W. The ideal and deviated data is given in Table 1. The content limit is assumed between 98.0% and 102.0% for all four groups. The value of AoA_a,_, tabulated in Table 1, has more drift toward lower or higher side of content limit of substance and it is justified as ΔAoA_a_= ΔAoA_p_×100/(100-W). The graph of AoA versus % water has been plotted and shown in (fig. 1).

**TABLE 1:** SIMULATED DATA REPRESENTING THE PROPAGATION OF ERROR DUE TO INACCURACY

	Ideal		Deviated		AoA			ΔAoA	
Group									
	AAI	W	AAI	W	AoA_i_	AoA_p_	AoA_a_	ΔAoA_p_	ΔAoA_a_
I	70	30	69.30	29.70	100.0	99.00	98.58	1	1.42
	75	25	74.25	24.75	100.0	99.00	98.67	1	1.33
	80	20	79.20	19.80	100.0	99.00	98.75	1	1.25
	85	15	84.15	14.85	100.0	99.00	98.83	1	1.17
	90	10	89.10	9.90	100.0	99.00	98.89	1	1.11
	95	5	94.05	4.95	100.0	99.00	98.95	1	1.05
II	70	30	70.70	30.30	100.0	101.00	101.43	1	1.43
	75	25	75.75	25.25	100.0	101.00	101.34	1	1.34
	80	20	80.80	20.20	100.0	101.00	101.25	1	1.25
	85	15	85.85	15.15	100.0	101.00	101.18	1	1.18
	90	10	90.90	10.10	100.0	101.00	101.11	1	1.11
	95	5	95.95	5.05	100.0	101.00	101.05	1	1.05
III	70	30	70.70	29.70	100.0	100.40	100.57	0.40	0.57
	75	25	75.75	24.75	100.0	100.50	100.66	0.50	0.66
	80	20	80.80	19.80	100.0	100.60	100.75	0.60	0.75
	85	15	85.85	14.85	100.0	100.70	100.82	0.70	0.82
	90	10	90.90	9.90	100.0	100.80	100.89	0.80	0.89
	95	5	95.95	4.95	100.0	100.90	100.95	0.90	0.95
IV	70	30	69.30	30.30	100.0	99.60	99.43	0.40	0.57
	75	25	74.25	25.25	100.0	99.50	99.33	0.50	0.67
	80	20	79.20	20.20	100.0	99.40	99.25	0.60	0.75
	85	15	84.15	15.15	100.0	99.30	99.18	0.70	0.82
	90	10	89.10	10.10	100.0	99.20	99.11	0.80	0.89
	95	5	94.05	5.05	100.0	99.10	99.05	0.90	0.95

**Fig. 1:**
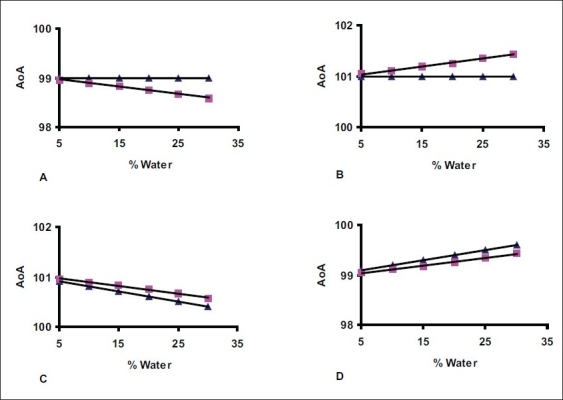
Plot of % AoA verses % Water content. The plots A, B and C, D represents constructive and destructive error of propagation, respectively. ▪ = AoAa, ▲ = AoA_p_

In normal analytical practice, the decision of acceptance or rejection of pharmaceutical use substance is based on AoA and its confidence interval. The mean value of AoA with confidence interval (CI) (i.e. AoA±CI) should completely fall in set range of content limit[4]. The simulated model-2 is prepared to understand the propagation of standard deviation error associated with AAI and W in AoA. The theoretical value of AAI and W of sodium citrate dihydrate is varied from 12.0% to 12.4% and 87.6% to 88.0%, respectively. The variation interval between two consecutive values of W and AAI is kept constant (i.e. 0.1%). The values of AAI and W are arranged in ascending and descending order respectively for destructive propagation. The both values of AAI and W are arranged in descending order for constructive propagation. The standard deviation for AAI and W is calculated for destructive and constructive mode of error propagation. The data of simulated model-2 is given in Table 2. The value of AoA_a_, tabulated in Table 2, is more inclined toward lower and higher content limit than AoA_p._ It is found that the standard deviation value is zero for destructive mode of error propagation through accepted and alternate formulas, which goes against theory of error propagation. In constructive mode of error propagation, the propagation of standard deviation of AAI and W to AoA_a_ is justified as SD_a_ = SD_p_×100/(100-W) (Eqn. 6). The substances for pharmaceutical use selected for experimental study were sodium dihydrogen phosphate dihydrate and sodium citrate dihydrate and anhydrous citric acid. The validity of Eqns. 5 and 6 is supported with experimentally determined values of AoA and its standard deviation of these three pharmaceutical use substances.

**TABLE 2:** SIMULATED DATA REPRESENTING THE PROPAGATION OF STANDARD DEVIATION ERRORS

Set	Destructive propagation		AoA_a_ = AoA_p_	Constructive Propagation		AoA_a_	AoA_p_
					
	AAI	W		AAI	W
1	87.6	12.4	100.00	88.0	12.4	100.46	100.40
2	87.7	12.3	100.00	87.9	12.3	100.23	100.20
3	87.8	12.2	100.00	87.8	12.2	100.00	100.00
4	87.9	12.1	100.00	87.7	12.1	99.77	99.80
5	88.0	12.0	100.00	87.6	12.0	99.55	99.60
Mean	87.8	12.2	100.00	87.8	12.2	100.00	100.00
Theoretical	87.8	12.2	-	87.8	12.2	-	-
Standard deviation	0.16	0.16	0.00	0.16	0.16	0.36	0.32

Sodium dihydrogen phosphate dihydrate, (SP) NaH_2_PO_4_·2H_2_O, MW 156.0, Sodium citrate dihydrate, (SC) C_6_H_5_Na_3_O_7_·2H_2_O, MW 294.1 and anhydrous citric acid, (CA) C_6_H_8_O_7_, MW 192.1 pharmaceutical grade substances obtained from Merck, India were used. Acetic acid glacial, CH_3_COOH, MW 60.05, Phenolphthalein, C_20_H_14_O_4_, MW 318.3 and 1-naphtholbenzein, C_27_H_20_O_3_, MW 392.5 were used of analytical grade of commerce. Pyridine free, Karl Fisher reagent solution of factor ~5 mg H_2_O/ml was used of commercially available grade. Potassium hydrogen phthalate C_8_H_5_KO_4_, MW 204.2 of certified volumetric standard was used.

The KF titrator, model-Mettler DL31, equipped with a dual platinum electrode and the autotitrator, model-Mettler DL67, equipped with a glass electrode were used. The water content was determined in six replicate of CA using 2.000 g and SC using 0.300 g. The method of analysis 2.5.12 was followed for water determination[5]. Loss on drying test was performed using 0.50 g at 130° for SP. The method of analysis 2.2.32 was followed for water determination of SP[6]. The assay test was performed in six replicates by using method described in European Pharmacopiea monographs of SP, SC and CA[7–9]. The experimental data of AAI and W were arranged in ascending order for constructive mode of error propagation. The AoA_a_ and AoA_p_ for each set of AAI and W were calculated. The arithmetic mean of AAI, W, AoA_a_ and AoA_p_ were calculated using Eqn.7 for arithmetic mean (Ā)= (x_1_+x_2_…x_i_)/n (Eqn.7). The standard deviation of AAI, W, AoA_a_ and AoA_p_ were calculated using Eqn.8 for standard deviation (SD)= [(Σx_i_−Ā)^2^/n-1]^½^ (Eqn.8). In Eqns.7 and 8 x_i_ is individual values and n is number of replicates. The values ΔAoA_a_ and ΔAoA_p_ was calculated as ΔAoA=100-AoA. The AoA_a_±CI_a_ and AoA_p_±CI_p_ were calculated using Eqn.9 for confidence interval(CI)=(t×SD)÷ (n)^½^ (Eqn.9) where t(student factor)=2.57 at 95% confidence interval and n=6[10]. All experimental data tabulated in Table 3 and 4.

**TABLE 3:** EXPERIMENTAL DATA OF AAI, W AND AoA

Substance	Set	AAI	W	AoA_p_	AoA_a_
Sodium dihydrogen phosphate dihydrate	1	76.04	23.17	99.21	98.98
	2	76.11	23.21	99.32	99.12
	3	76.20	23.22	99.42	99.25
	4	76.22	23.27	99.49	99.33
	5	76.27	23.32	99.59	99.47
	6	76.33	23.57	99.90	99.87
Mean	-	76.20	23.29	99.49	99.33
Standard deviation	-	0.1	0.15	0.24	0.31
Sodium citrate dihydrate	1	87.67	11.50	99.17	99.06
	2	87.67	11.65	99.32	99.24
	3	87.79	11.75	99.54	99.48
	4	88.03	11.78	99.81	99.78
	5	88.13	11.92	100.05	100.06
	6	88.42	12.06	100.48	100.55
Mean	-	87.95	11.78	99.73	99.69
Standard deviation	-	0.30	0.20	0.49	0.55
Anhydrous Citric acid	1	99.5	0.0974	99.60	99.60
	2	99.5	0.0980	99.60	99.60
	3	99.8	0.0984	99.90	99.90
	4	99.7	0.1002	99.80	99.80
	5	100.1	0.1029	100.20	100.20
	6	100.3	0.1062	100.41	100.41
Mean	-	99.82	0.10	99.92	99.92
Standard deviation	-	0.34	0.00	0.33	0.33

Based on water content result of SP the extent of propagation of standard deviation error and magnitude of drift through accepted formula was predicted to 1.30 i.e. [100/(100-W)]=100/(100–23.29)=1.30. The experimental value of extent of propagation of standard deviation error was found to 1.29 i.e. (SD_AoAa_/SD_AoAp_)=0.31/0.24=1.29. The experimental value of magnitude of drift was found to 1.31 i.e. (100-AoA_a_)/(100-AoA_p_)=100-99.33/100-99.49=1.31

The similar trends of observations were found for SC and CA. The predicted and experimental value of extent of standard deviation error propagation to AoA_a_ through accepted formula was comparable for SP and SC. The predicted and experimental magnitude of drift in accepted formula was comparable for SP and SC. There was no impact on drift of AoA_a_ and it's standard deviation for CA because the value of 100/(100−0.1) was almost equals to 1. The data related to ΔAoA_a,_ΔAoA_p_, SD_AoAa_ and SD_AoAp_ tabulated in Table 4. Experimentally, it was proved that the extent of propagation of errors obtained by industry-accepted formula was found higher by a factor 100/(100-%water) in comparison with alternate formula. The cause of higher standard deviation and inaccuracy has been identified in industry-accepted formula. The drift and propagation of errors should be considered during setting specification limit of substances containing higher amount of water.

**TABLE 4:** EXPERIMENTAL - PROPAGATED ERRORS DATA

Parameter	Sodium dihydrogen phosphate dihydrate	Sodium citrate dihydrate	Anhydrous Citric acid
ΔAoA_a_	0.67	0.31	0.08
ΔAoA_p_	0.51	0.27	0.08
ΔAoA_a_/ΔAoA_p_	1.31	1.15	1.00
SD_AoAa_	0.31	0.55	0.33
SD_AoAp_	0.24	0.49	0.33
SD_AoAa_/SD_AoAp_	1.29	1.12	1.00
100/(100-W)	1.30	1.13	1.00
AoA_a_ ± CI_a_	99.33 ± 0.33	99.69 ± 0.58	99.90 ± 0.35
AoA_p_ ± CI_p_	99.49 ± 0.25	99.73 ± 0.51	99.90 ± 0.35
